# A locus-dependent mixed inheritance in the segmental allohexaploid sweetpotato (*Ipomoea batatas* [L.] Lam)

**DOI:** 10.3389/fpls.2024.1398081

**Published:** 2024-05-28

**Authors:** Ming Gao, Tien Hua, Genhua Niu, Joe Masabni, Willie Dewalt

**Affiliations:** ^1^Cooperative Agricultural Research Center, College of Agriculture, Food and Natural Resources, Prairie View A&M University, Prairie View, TX, United States; ^2^AgriLife Research and Extension Center at Dallas, Texas A&M University, Dallas, TX, United States

**Keywords:** segmental-allohexaploid sweetpotato, digital-quantitative PCR genotyping, homoeolog-types, F2-population, mixed inheritance

## Abstract

Two interrelated aspects of the sweetpotato genome, its polyploid origin and inheritance type, remain uncertain. We recently proposed a segmental allohexaploid sweetpotato and thus sought to clarify its inheritance type by direct analyses of homoeolog segregations at selected single-copy loci. For such analyses, we developed a digital quantitative PCR genotyping method using one nondiscriminatory and three discriminatory probes for each selected locus to discriminate and quantify three homoeolog-differentiating variation types (homoeolog-types) in genomic DNA samples for genotype fitting and constructed a F2 population for segregation analyses. We confirmed inter-subgenomic distinctions of three identified homoeolog-types at each of five selected loci by their interspecific differentiations among 14 species in Ipomoea section batatas and genotyped the loci in 549 F2 lines, selected F1 progenies, and their founding parents. Segregation and genotype analyses revealed a locus-dependent mixed inheritance (disomic, polysomic, and intermediate types) of the homoeolog-types at 4 loci in the F2 population, displaying estimated disomic-inheritance frequencies of 0, 2.72%, 14.52%, and 36.92%, and probably in the F1 population too. There were also low-frequency non-hexaploid F1 and F2 genotypes that were probably derived from double-reduction recombination or partially unreduced gametes, and F2 genotypes of apparent aneuploids/dysploids with neopolyploid-like frequencies. Additional analyses of homoeolog-type genotypes at the 5 loci in 46 lines from various regions revealed locus-dependent selection biases, favoring genotypes having more of one homoeolog-type, i.e. more of di- or homogenized homoeolog-type composition, and one-direction ploidy trending among apparent aneuploids/dysploids. These inheritance features pointed to an evolving segmental allohexaploid sweetpotato impacted by selection biases.

## 1 Introduction

Sweetpotato (*Ipomoea batatas* (L.) Lam.) is an important crop for food, feed, and industrial raw materials. Recent research advances in sweetpotato genomics and genetics have improved our understanding of its genome and inheritance (Yan et al., 2022). However, two interrelated and critical aspects of the sweetpotato genome, its polyploid origin and inheritance type, remain uncertain and controversial. Most recent relevant studies continued a long-standing debate on its auto- or allohexaploid origin. A deduced weak preferential and near-random pairing among homologous chromosomes in a recent multi-locus mapping study led to the conclusion of a hexasomic inheritance in an autohexaploid sweetpotato (Mollinari et al., 2020). Several other studies simply assumed an autohexaploid sweetpotato for mapping or modeling (Shirasawa et al., 2017; Da Silva Pereira et al., 2020; Yamamoto et al., 2020; Chen et al., 2021). However, in a genome-wide eQTLs analysis, homologous and homoeologous SNPs were distinguished using the *Ipomoea trifida* genome sequence as a subgenome reference (Zhang et al., 2020), essentially assuming an allohexaploid sweetpotato for the analysis. All these mapping studies were based on analyses of bi-type alleles with two additional unspoken assumptions, i.e., no double-reduction recombination (Jones, 1967) and no aneuploidy or dysploidy in the mapping populations, which are unlikely to be true considering known reproductive behaviors in polyploids (Ramsey and Schemske, 2002; Mayrose and Lysak, 2021). Moreover, a tetraploid *Ipomoea batatas* (Yan et al., 2021) and a newly identified tetraploid species (Munoz-Rodriguez et al., 2022), *Ipomoea aequatoriensis*, have been proposed to be a direct progenitor to sweetpotato, implying an interspecific hybridization speciation (allohexaploid) of sweetpotato. We recently proposed an interspecific hybridization speciation of sweetpotato and a post-polyploid genome evolution to a segmental allohexaploid based on distinguishable homoeolog differentiations at many single-copy loci and a hybridization-network phylogeny reconstruction (Gao et al., 2020). This conclusion was further supported by a recent chromosome painting study (Sun et al., 2022).

We thus sought to clarify the inheritance type in a probable segmental allohexaploid sweetpotato by detailed analyses of segregations of three homoeolog-differentiating variation types (homoeolog-types) at selected single-copy loci among those identified in our previous study (Gao et al., 2020). For such analyses, we developed a digital quantitative PCR (dqPCR) genotyping method using LNA (locked nucleic acid)-FRET probes to accurately discriminate and simultaneously quantify three homoeolog-type targets in genomic DNA samples for fitting their ratios (i.e., genotypes) and a currently only F2 population in sweetpotato for segregation analyses. We confirmed the inter-subgenomic distinctions of the three homoeolog types at each of the five selected loci by their interspecific differentiations among 14 species in Ipomoea section batatas. We then genotyped the 5 loci in 549 F2 lines, 20 selected F1 progenies, and their founding parents, and analyzed the homoeolog-type inheritance in the two generations. We further evaluated the effects of breeding selections on the homoeolog-type compositions at the 5 loci in 46 sweetpotato lines from various cultivation regions. These analyses revealed quite different inheritance patterns in sweetpotato than what is currently assumed or concluded.

## 2 Materials and methods

### 2.1 Plant materials

Thirty-seven cultivars from worldwide growth regions and 13 crop-relative species in Ipomoea Section Batatas, including five accessions of *Ipomoea trifida*, were obtained from the USDA-ARS Plant Genetic Resources Conservation Unit, Griffin, GA (https://npgsweb.ars-grin.gov/gringlobal/site.aspx?id=22). Their accession numbers and regions of origin were listed in the **Supplementary Data S8** and **S11**. Another six cultivars, including Boniato, Japanese purple, O’Henry, Murasaki-1, Covington, and Bonita, were originally from a commercial source (https://www.sweetpotatoplant.com). The CH-purple and CH-vegie (farmers’ labels) lines were derived from locally grown farm varieties. All other sweetpotato lines are in-house breeding lines. All hybridization and R5 self-crossing were conducted using open-field pollination between the respective parental lines and seeds harvested from the maternal lines.

### 2.2 DNA extraction

All genomic DNA samples were prepared from young leaves using the *Quick*-DNA Plant/Seed Miniprep Kit (https://www.zymoresearch.com) and initially quantified by UV spectrophotometry. Concentrations of all diluted genomic DNA samples for dqPCR were further calibrated using the Qubit dsDNA HS Assay Kit (ThermoFisher Scientific, CA).

### 2.3 Genomic-amplicon sequences and alignment

Sequences of genomic amplicon variants at five selected single-copy loci from two sweetpotato lines and of reference homologs from *I. tenuissima* were from a previous study (Gao et al., 2020). Sequences of the reference homologs from *I. trifida* were from the genome assembly of the NSP306 accession (http://sweetpotato.uga.edu/gt4sp_download.shtml). The Geneious software package (Kearse et al., 2012) was used for sequence alignments.

### 2.4 dqPCR and homoeolog-type genotyping

The two-channel QuantStudio™ 3D Digital PCR system was first used to screen and test the Affinity Plus™ (Integrated DNA Technologies, IA) LNA-FRET qPCR probes for specificity and signal strength, for optimal dqPCR conditions, and for probe combination compatibility. Manufacturer’s guidelines were followed for the preparation of reaction mixes, chip loading and amplification, and chip imaging. Briefly, a reaction mixture of 34.8 µl for two chips includes 17.4 µl of dqPCR master mix (2X), 1.7 µl of 20X primer/probe mix (0.4, or 0.6, or 0.9 µM each of two PCR primers and 0.1 or 0.15 µM each of two probes in final concentrations), 3.5 µl diluted genomic DNA, and 12.2 µl of water. The final genomic DNA concentration varied from 200 to 1900 pg/µl, depending on the probe-target concentration in a genomic DNA sample. Each chip (20K Chip Kit V2) was loaded with 14.5 µl of the reaction mix using a chip loader and amplified using the manufacturer’s recommended protocol with annealing temperatures at 56, 58, 59, 60, 62, and 64^°^C for optimization for different primer and probe combinations.

The optimized concentrations and annealing temperatures of the screened primers and probes for all 5 loci were applied to dqPCR using the five-channel QIAcuity Digital PCR System. All genotyping dqPCR contained 1X of the QIAcuity Probe PCR master mix, 0.625 µM each of two PCR primers, 0.125 µM each of four probes (A, B, C, and control types), and varying amounts of diluted genomic DNA (depending on probe-target concentrations and DNA samples) in a final 40 µl volume per well in a QIAcuity nanoplate (24-Well and 26k partitions). To minimize quantitation errors caused by variations of DNA concentration and plastid and mitochondrial DNA contents in genomic DNA samples, identical volumes (20 μl in most cases) of the same genomic DNA dilution were usually taken for dqPCRs with different primer and probe combinations for genotyping different loci in a sweet potato line or crop-relative species. The cycling conditions are as follows: 1 cycle of 95°C for 2 min, 40 cycles of 95°C for 15 sec, and 60 or 64°C for 30 sec. The combined annealing and extension temperature of 64°C was only for the primers and probes for the Ibit12692, and 60°C for all others.

### 2.5 Statistical analyses

The Statulator (https://statulator.com) and the XLSTAT-Life Science package as a Microsoft Excel add-on were used for the Z-value-based chi-square and the multinomial goodness of fit tests, respectively, for comparing observed genotype frequencies in the F2 population with their expected frequencies under polysomic inheritance.

## 3 Results

### 3.1 Defining and probing homoeolog-types at selected loci

**Figure 1:**
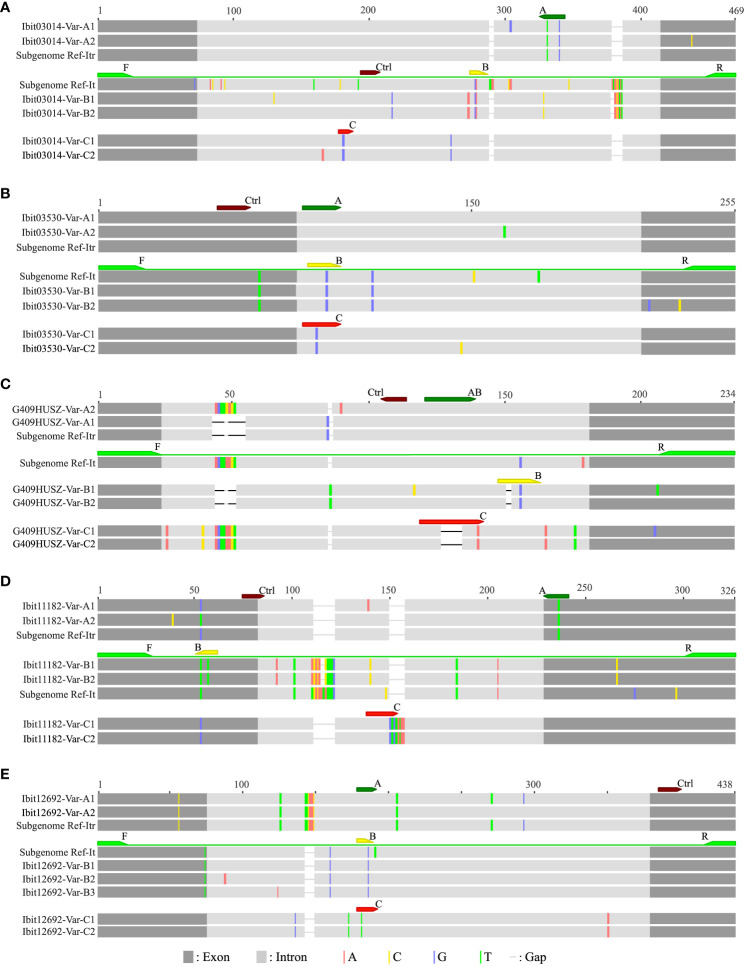
Graphic illustrations of dqPCR-genotyping primers and probes in targeted regions at five selected single-copy loci. The alignments **(A–E)** illustrate the dqPCR-targeted regions at the five selected single-copy loci with graphically represented amplicon variants (‘-Var-’ in the identifiers) from two sweetpotato lines and corresponding reference homologs from *I. tenuissima* and *I. trifida*, which were extracted from those of longer ones in our previous study (Gao et al., 2020), and from the assembled I. trifida (NCNSP0306) genome sequence (Wu et al., 2018), respectively. The identifiers for the five loci (Ibit03014, Ibit03530, G409HUSZ, Ibit11182 and Ibit12692), which were the genomic sequence identifiers from our previous targeted genome sequencing of two sweetpotato lines (Gao et al., 2020), were used as the prefixes of the identifiers for the group of genomic variants at the corresponding loci. The corresponding reference homologs from *I. tenuissima* and *I. trifida* at each of the five loci were labeled as Subgenome Ref-It and Subgenome Ref-Itr, respectively. The color-highlighted sequence variations were those against the consensus in each alignment. The positions and relative sequence length of the dqPCR-genotyping primer pairs (F/R) and a set of four probes (A, B, C and Ctrl for the control) were marked by correspondingly colored arrows.

For analyses of homoeolog inheritance in sweetpotato, we used three types of inter-subgenomic sequence variations at selected single-copy loci to distinguish and mark homoeologs; hence, we named them homoeolog-types, and we developed LNA-FRET probes to accurately discriminate and simultaneously quantify three homoeolog-types in dqPCR for genotyping. We initially selected ten single-copy loci among those identified in our previous study (Gao et al., 2020), but were only able to develop a set of four multiplexable probes (one non-discriminatory control and three discriminatory ones) each for five of them (**Supplementary Table S1**). **Figure 1** graphically illustrates the defining and dqPCR-probing of the A, B, and C homoeolog-types at the five selected loci. The A, B, and C homoeolog-types each at four of the loci except the G409HUSZ were distinguished by their interspecific sharing (partial for the B homoeolog-type at the Ibit11182) in reference homologs from *I. trifida* (NCNSP0306), *I. tenuissima*, or in neither of the references, respectively, and targeted by correspondingly named probes. While the B and C homoeolog-types at the G409HUSZ were likewise distinguished and targeted by specific probes, the A homoeolog-type at the locus was indirectly defined and resolved by the probe AB minus B targets. The control probe for each of the 5 loci targeted a highly conserved exon or intron region for detecting all homoeolog-types. The 5 loci probably represent four homoeologous groups in sweetpotato: the G409HUSZ, the Ibit03530, and the Ibit03014 each for one, and the Ibit11182 and the Ibit12692 for another one, as their corresponding homologs in *I. trifida* are located on chromosomes 6, 7, 12, and 14, respectively.

### 3.2 dqPCR genotyping homoeolog-types

**Figure 2:**
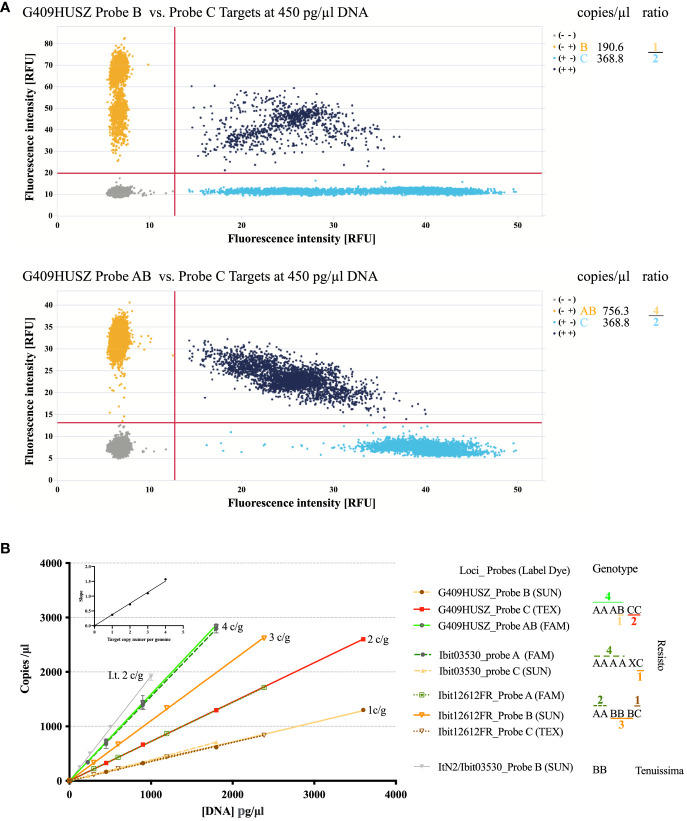
Illustration of the dqPCR method for genotyping homoeolog-types in sweet potatoes. **(A)** 2-D scatter plots of the G409HUSZ probes B *vs.* C and AB *vs.* C target signals from one dqPCR reaction with the Resisto genomic DNA at 450 pg/ml. The probe target concentrations from the dqPCR and fitted ratios of the homoeolog-types were listed on the right. **(B)** Linear responses of the probe-target concentrations to varying amounts of genomic DNA in dqPCRs and dependence of the response-line slopes on the per-genome target copy numbers (c/g). Target concentrations of three sets of homoeolog-type probes (for 3 loci) detected in four sets of dilutions of the same Resisto genomic DNA sample and those of the sweetpotato Ibit03530 B-type probe in four dilutions of an *I*. *tenuissima* genomic DNA sample (ItN2/03503-Probe B) were plotted against corresponding genomic DNA concentrations in dqPCR reactions. Target concentrations of corresponding control probes for the 3 loci were not included in the plot. The Ibit03530_probe B did not detect any target, while the control probe for the Ibit03530 detected an additional homoeolog (X) that was null-signaled to the A, B, and C-type probes for the locus. The numbers of A (or AB), B, and C homoeolog-type in the genotypes were from fitted integer ratios of the corresponding probe-target concentrations measured from the same dqPCR, which were marked with corresponding colors for the probes and noted above or under the homoeolog-type signs. The averaged slopes for each of the four groups of the probe-target *vs.* DNA concentration lines were plotted against their corresponding 1 to 4 per-genome probe-target copy numbers in the embedded graph.

We developed a dqPCR genotyping method to avoid distortions of highly lopsided ratios of the homoeolog-types (e.g., 4 or 5 to 1, or even larger) at some loci by disproportional amplification and to effectively resolve aneuploid and dysploid genotypes. **Figure 2** illustrates the principles and examples of genotyping homoeolog-types by dqPCR. The two 2D-scatterplots from genotyping the G409HUSZ in the Resisto cultivar by a 4-probe dqPCR (the control probe is not shown) exemplified clear target discrimination by the three probes. **Figure 2B** shows linear responses of the probe-target concentrations to a wide range of DNA concentrations in dqPCR genotyping of 3 loci in Resisto with three corresponding sets of probes and the Ibit03530-homolog locus in *I. tenuissima* with the Ibit03530 probe B. More importantly, it also demonstrated equal slopes of the response lines for different probe-targets having the same per-genome copy numbers and a proportional linear relationship between the slopes for the response lines and the per-genome copy numbers of the probe-targets when the same Resisto DNA sample was used in the dqPCRs. In other words, in dqPCR genotyping of different loci using the same genomic DNA sample, the slopes for the per-genome single-copy probe-target vs. DNA concentration lines at the same or different loci should be statistically equal. For all dqPCR genotyping (**Supplementary Data S8**, **S11**), we calculated slopes for the per-genome single-copy target averaged from those of the A, B, and C probe-targets (S_ABC_) and of the control probe-target (S_Ctrl_) (i.e., the total concentration of the three probe-targets or the control probe-target/the product of the total number of the homoeolog-types in the called genotype times the DNA concentration in the dqPCR). If genotypes were called correctly by fitted ratios of probe-targets from their concentrations measured in multiple dqPCRs that genotype different loci using the same DNA sample, the differences between the S_ABC_ and S_Ctrl_ calculated using the total numbers of the homoeolog-types in the called genotype(s) for the same or different locus should be statistically insignificant.

We called genotypes from measured probe-target concentrations using combinations of three methods. First, we used the smallest and uneven-numbered integer ratio that the measured A (or AB), B, and C probe-target concentrations from the same dqPCR could best fit to directly call the homoeolog-type ratio in a genotype. **Figure 2** demonstrated such genotype calling from a best-fitted 2:3:1 ratio of A:B:C probe-target concentrations at the Ibit12692 and a 4:1:2 ratio of AB:B:C at the G409HUSZ in a Resisto DNA sample to the AABBBC and AAABCC genotypes at the 2 loci. The chi-square goodness-of-fit p-value for such fitted homoeolog-type ratios in so-called genotypes was all larger than 0.80. Such genotypes were further confirmed by the insignificant difference between the corresponding S_ABC_ and the S_Ctrl_ at the loci. Secondly, we applied the principle of statistically equal S_ABC_ and S_Ctrl_ from the same dqPCR to resolve a genotype having null-signaled homoeolog-types. On average, a statistically significant discrepancy between the S_ABC_ and S_Ctrl_ from the same dqPCR is >0.06 for the difference caused by one per-genome target copy in sweetpotato. For example, at the Ibit03530 in Resisto, the S_ABC_ vs. S_Ctrl_ for the fitted 4:0:1 ratio of A:B:C probe-targets was 0.349 vs. 0.431 both by per-genome five target copies, having a statistically significant difference of 0.082, but was 0.349 vs. 0.359 by five and six per-genome target copies, respectively, to lead to an insignificant difference of 0.01. This indicated that the control probe detected one more per-genome target copy that was null-signaled to all three discriminatory probes, so that the genotype should be AAAACX (the null-signaled) instead of AAAAC. This ‘X’ could be a mutated version of either one of the three homoeolog-types, which had additional variations that nullified the probe detection. Additionally, spontaneous mutations in one or more of the control probe targets could also nullify the signal, leading to a smaller S_Ctrl_ than the corresponding S_ABC_ by the difference from one (or more) per-genome target copy. We detected two such rare cases at the Ibit11182 in two sweetpotato lines (PI318846 and PI 595873 in the **Supplementaryray Data S11**) among more than 2500 called genotypes. The homoeolog-type identities for both types of the null-signaled ‘X’ (i.e., mutated A, B, or C) cannot be resolved without sequencing. However, their very low occurrence frequencies in a large population make them statistically insignificant for ratio analyses of the genotypes in the population (e.g., segregation analyses in this study). Lastly, for fitted probe-target ratios of equal proportions or undefined ones (1:1:1, 1:1:0, and 1:0:0, etc.), we determined the total numbers of homoeolog-types in called genotypes by their fitting to yield a S_ABC_ and a S_Ctrl_ having no statistically significant differences between each other and between the two slopes and those from genotyping of other loci using the same DNA sample, i.e., ≤ 0.06. All the genotypes called using the second and third methods, and those of non-hexaploid types in this study, were further confirmed by at least three repeated dqPCR genotypings using independently isolated genomic DNA samples.

### 3.3 Interspecific differentiation of homoeolog-types

**Figure 3:**
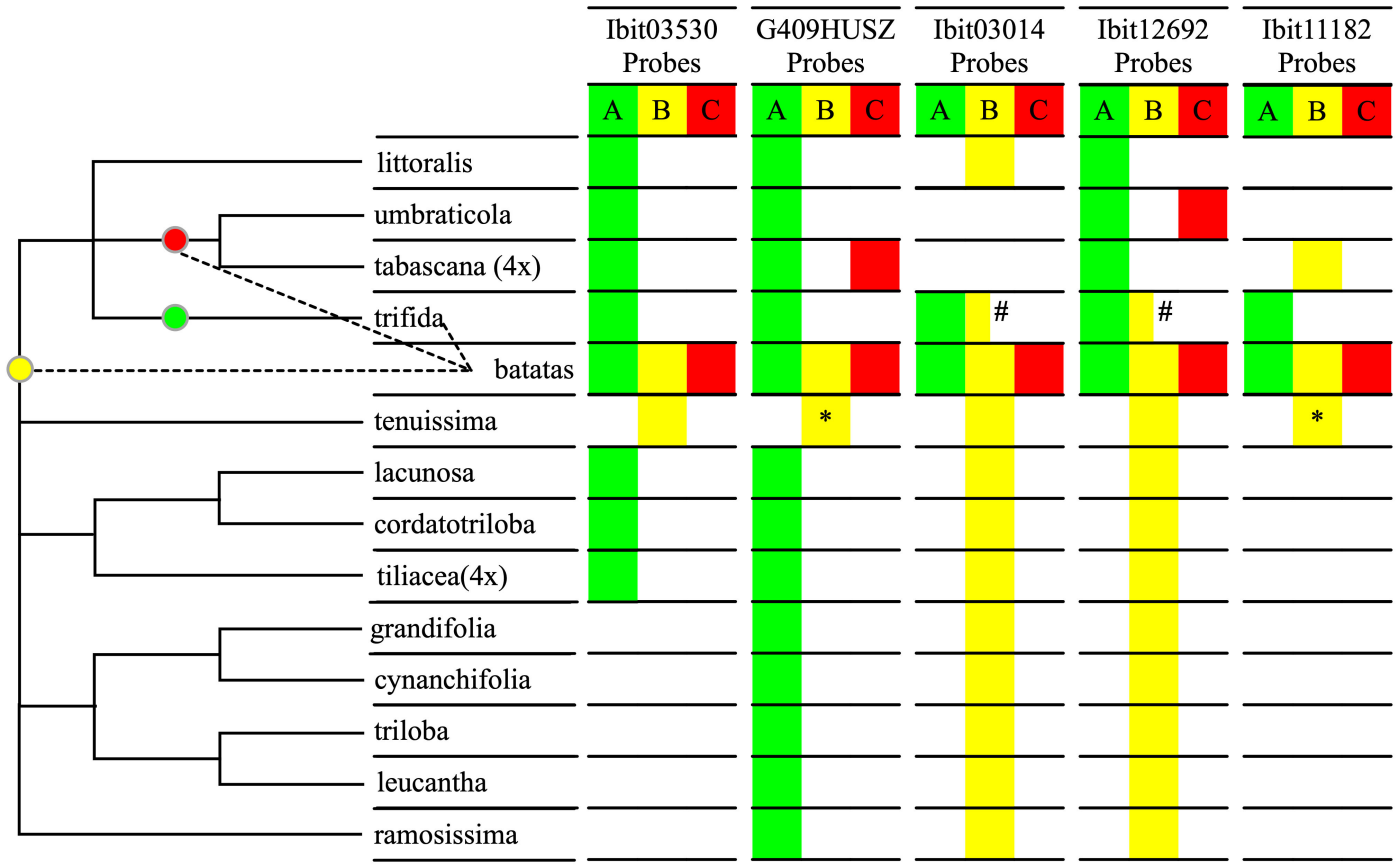
Interspecific differentiation of the three homoeolog-types. The table summarizes interspecific sharing of the A, B, C homoeolog-types at each of the 5 loci by homologs in the fourteen species in the Ipomoea Section Batatas using three corresponding color fills based on dqPCR genotyping results, except for the two with asterisks. The two yellow fills with asterisks indicate partial matches of the probe-covered B homoeolog-types at the G409HUSZ and the Ibit11182 in sweetpotato with those in *I. tenuissima* based on their sequences from our previous study (Gao et al., 2020). The half-yellow fills with the # sign indicate that the B homoeolog-type at the Ibit03014 and the Ibit12692 loci were detected in only one of five genotyped *I. trifida* accessions. The cladogram on the left was constructed based on the grouping of the 14 species by the distribution patterns of the homoeolog-types and on their plastome-based phylogeny (Sun et al., 2019). The three colored dots on the cladogram indicate probable positions of source species nodes for the three homoeolog-types in sweetpotato in relation to the thirteen of its closest relatives.

We sought to further confirm the inter-subgenomic distinction of the three homoeolog-types at the 5 loci by their interspecific differentiations. We genotyped the 5 loci in 14 species of the Ipomoea section batatas, including five *I. trifida* accessions, using the dqPCR method with the five sets of probes, and summarized the results (**Supplementary Data S8**) using a qualitative color map in **Figure 3**. The distribution patterns of the homoeolog-types at the 5 loci clearly divided 12 of the 14 species into three groups, and sweetpotato and *I. tenuissima* by themselves. We integrated this grouping and the relationships of these species in a plastome-based phylogenetic tree (Sun et al., 2019) into a cladogram to identify relationships between the sources of the A, B, and C homoeolog-types and the 14 species. Across the 5 loci, *I. trifida* is the only species that shared all the A homoeolog-types at the five sweetpotato loci and exclusively at the Ibit03014 and the Ibit11182, which is consistent with it being a progenitor of sweetpotato. *I. tenuissima* shared the complete homoeolog-types at 3 loci and exclusively at one of them, and partial ones (by sequences) at another two, which is consistent with it being closest to a second progenitor for the B homoeolog-type. *I. umbraticola* and *I. tabascana* each shared the C homoeolog-types exclusively at the two different loci, indicating that they were among the closest to the third progenitor of sweetpotato. Vertically among the species, the A, B, and C homoeolog-types at the G409HUSZ and the Ibit12692, and the A and B at the Ibit03530 and the Ibit11182 (no detectable C type) indeed represented interspecific differentiations among homologs. The B homoeolog-types at the Ibit03014 and the Ibit12692 were detected as an apparent BBB triploid genotype in the PI618966 accession of *I. trifida*, but were also shared in another 10 and 9 related species, respectively. Thus, these B homoeolog-types at the loci in extant *I. trifida* accessions were not necessarily the sources of those in sweetpotato. The distribution patterns of the five sets of homoeolog-types indicated that they probably originated from *I. trifida* and two of its unidentified closest-relative species, as marked on the cladogram.

### 3.4 Construction of the F2 population and notable F1 genotypes

**Figure 4:**
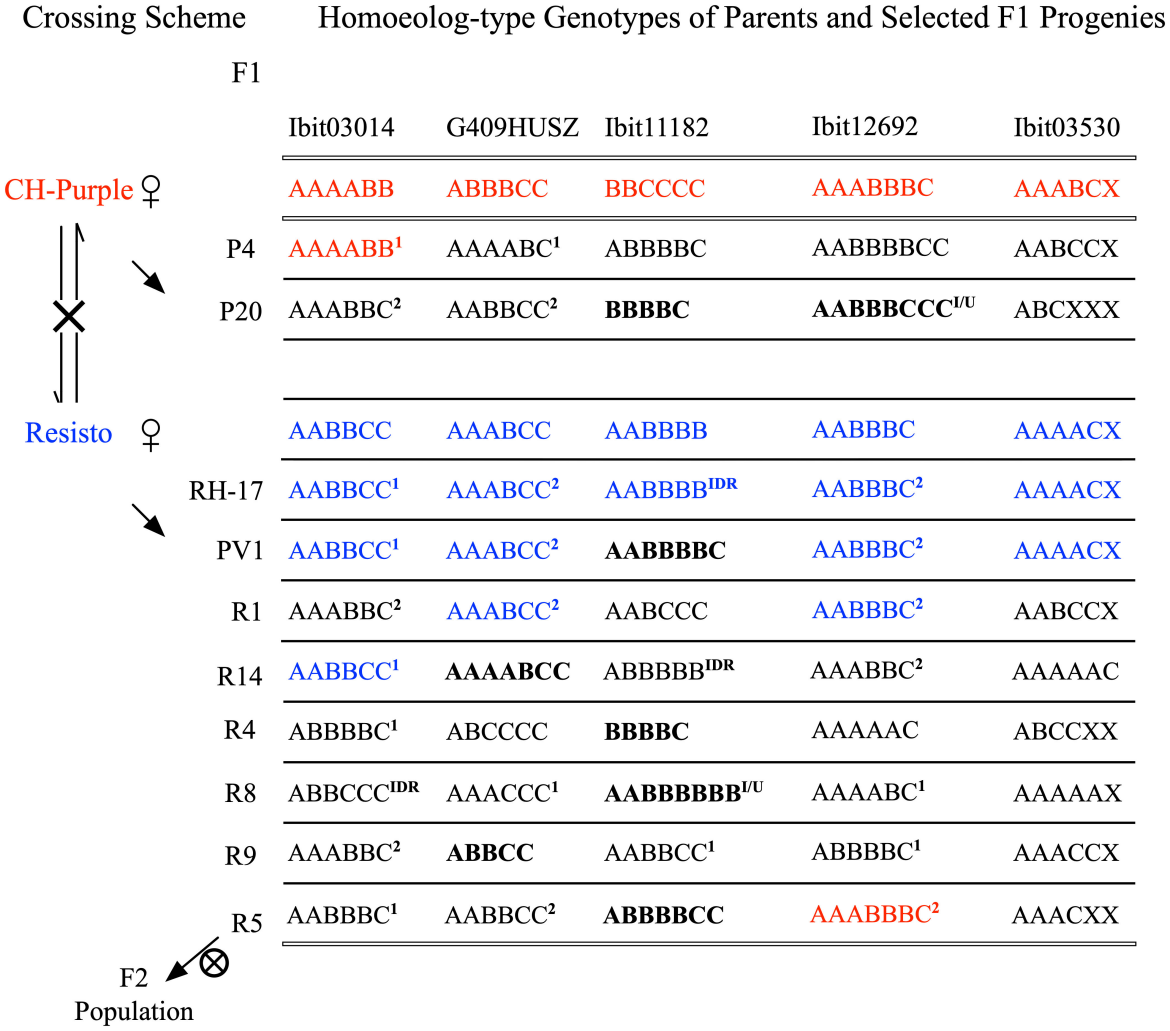
Crossing scheme for generating the F2 population and summary of some notable F1 genotypes. Reciprocal crosses between the Resisto and CH-Purple lines yielded two groups of F1 progenies, separately mothered by the two lines. One of the selected Resisto-mothered F1 lines, the R5, is self-fertile and has produced a large F2 population by natural self-pollination. Several other F1 progenies with notable homoeolog genotypes were listed. The two sets of parental genotypes at the 5 loci were highlighted with red and blue letters. The bold-letter genotypes marked apparent aneuploids or dysploids. The “X” in genotypes at the Ibit03530 loci indicates a homoeolog type that was null-signaled to the A, B, C probes but detected by the control probe. ^IDR^: genotypes with a “Identical-by-Double-Reduction” pair of the noted B or C homoeolog-type. ^1^: genotypes involving one gametic genotype from preferential paring of the same homoeolog-types (i.e., AA, BB, or CC). ^2^: genotypes involving both gametic genotypes from preferential paring of the same homoeolog-types (i.e., AA, BB, or CC) or the ones from random pairing of all the 6 or 7 homoeolog-types.

We constructed a very large F2 population of 600 germinated lines and additional more than 3000 seeds from natural self-pollinations of a self-fertile F1 line, the R5, serendipitously identified among more than 650 F1 lines. **Figure 4** illustrates the crossing scheme leading to the F2 population and notable homoeolog-type genotypes at the 5 loci in selected F1 lines among 20 genotyped ones (3 CH-Purple-mothered and 17 Resisto-mothered) and in their parents (**Supplementary Data S9**). These F1 genotypes, albeit in small numbers, displayed three patterns. First, high frequencies of maternal genotypes (blue-colored) stand out at the 5 loci in the Resisto-mothered F1 lines, three each at 3 loci, and two and one at the other 2 loci. Most of these Resisto-maternal F1 genotypes and many others (^1^ or ^2^ marked) predictably involved one or both gametic genotypes that could be derived from either preferential pairing of the homologous homoeolog-types (i.e., preferential AA, BB, or CC pairing) or from complete random paring of all six or seven homoeolog-types under bivalent configuration. Frequencies of such partial or full dual-sourced genotypes could be used to evaluate the inheritance types at the loci. The high frequencies of the full dual-sourced Resisto-maternal genotypes at the G409HUSZ (3 AAABCC/17 genotypes) and the Ibit12692 (4 AABBBC/17 genotypes) (**Supplementary Table S2**). were probably due to high proportions of disomic inheritance in a mixed inheritance, as they were much closer to those expected from a preferential pairing than to those from a complete random pairing of the homoeolog-types, 25% vs. 10.5% and 16.7% vs. 4.2%, respectively. At Ibit03014, the frequency of the partial dual-sourced Resisto-maternal genotype (AABBCC) was significantly higher than expected from a hexasomic inheritance (3/17 vs. expected 3/40), indicating the additional contribution of a gametic genotype from a preferential pairing. But the frequency of the full dual-sourced genotype (AAABBC) at the Ibit03014 was significantly lower than expected from disomic and hexasomic inheritances (2/17 vs. 17/17 and 1/5), probably resulting from biased selection.

Second, there were several genotypes (bold-letter marked) of apparent ploidy unexpected from either disomic or hexasomic inheritance: the 5-ploids at 2 loci in three lines, the 7-ploids at 2 loci in three lines, and the 8-ploids at 2 loci in two lines. The co-occurrence of the 5- and 7-ploid genotypes with paired loss/gain of one ‘ploid’ at the G409HUSZ and the Ibit11182 among the Resisto-mothered F1 lines indicated that these genotypes were probably aneuploids or dysploids, rather than copy number variations from random deletions or cis-duplications. We thus used the ‘apparent ploid’ level to describe these non-hexaploid genotypes. Lastly, three genotypes predictably carried “identical-by-double-reduction” (IDR) homoeolog-type pairs. The AABBBB and ABBBBB genotypes from BBCCCC x AABBBB, or the ABBCCC from AAAABB x AABBCC, must have involved a ‘BBB’ gametic genotype from the paternal BBCCCC or a ‘CCC’ from the maternal AABBCC, respectively, which could have been derived only from a normally segregated B or C plus a pair of B’s or C’s that were brought into the same gamete from those on two sister chromatids by double-reduction, respectively. Formation of the 8-ploids (I/U) at the Ibit11182 in the R8 and at the Ibit12692 in the P4 and P20 lines could have involved either gametes carrying an IDR B or C homoeolog-type pair or gametes with partially unreduced BB or CC pairs from unbalanced segregations.

### 3.5 A locus-dependent mixed inheritance and unexpected genotypes in the F2 population

**Figure 5:**
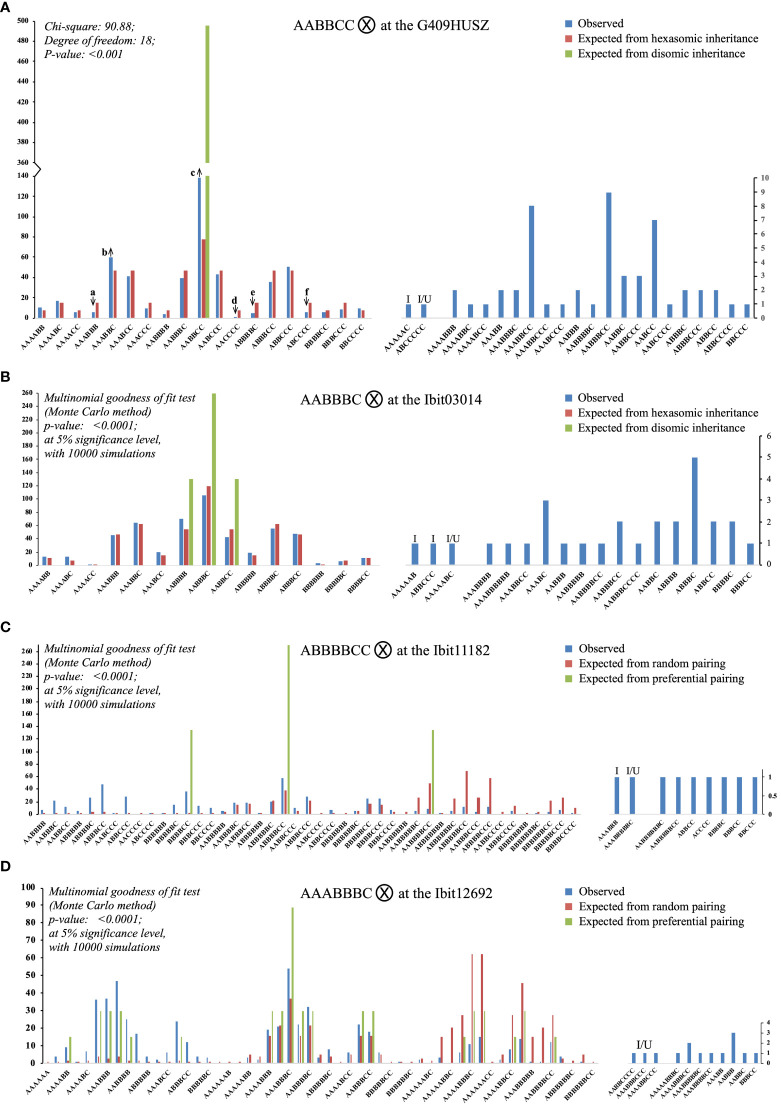
Segregations of the three homoeolog-types each at four selected loci in the F2 population. Each of the **(A–D)** charts summarizes the observed counts of homoeolog-type genotypes at a locus in the F2 population and compares them with those of the same genotypes expected from either preferential pairing of homologous homoeolog-types (i.e., AA, BB, and CC pairing first, disomic inheritance) or complete random pairing among all homoeolog-types (i.e., polysomic inheritance) under bivalent configuration. The up and down arrows in **(A)** indicate that an observed proportion was significantly overrepresented or underrepresented, respectively, as compared to its corresponding expected proportion, with the Z-test p-values at 0.014, 0.036, <0.001, 0.015, 0.007, and 0.014 (a to f). I: genotypes carrying an “identical-by-double-reduction” pair of a homoeolog-type. I/U: genotypes either with an “identical-by-double-reduction” pair of a homoeolog-type or involving one or two genotypes of unreduced gametes.

We genotyped the 5 loci in 549 F2 progenies (**Supplementary Data S10**) and analyzed homoeolog-type segregations at four of them. We skipped the segregation analysis at the Ibit03530 to avoid complications of the null-signaled homoeolog-type but used the genotypes expectedly lacking the B homoeolog-type from the selfing of the AAACX parental genotype to confirm no contamination in the population. The adequacy of the genotyped population size for non-skewed genotype representations was confirmed by statistically equal ratios of the A:B:C homoeolog-types in 549 F2 genotypes at each of the loci and in the corresponding parental genotype. The ratios of the total A:B:C in 549 genotypes each at the G409HUSZ, the Ibit03014, the Ibit11182, and the Ibit12692 were 1151:1086:1073 (1:1:1), 1115:1647:526 (2:3:1), 507:2050:1117 (1:4:2), and 1484:1654:550 (3:3:1), which were statistically equal to those in the corresponding parental genotypes of AABBCC, AABBBC, ABBBBCC, and AAABBBC, respectively. We compared the observed genotypes and their counts at each of the 4 loci in the 549 F2 lines with those expected from either preferential pairing of homologous homoeolog-types (i.e., disomic inheritance) or complete random pairing among all the 6 or 7 homoeolog-types (i.e., polysomic inheritance), both under bivalent configuration without double reduction (**Figure 5**; **Supplementary Tables S3**-**S6**).

There were three identical categories of segregated F2 genotypes at each of the 4 loci. The first included genotypes (the left half of the bipartite charts in **Figure 5**) matching in classes to those expected from random pairing of all the homoeolog-types under bivalent configuration (i.e., polysomic inheritance), i.e., 19 and 15 hexaploid genotypes out of the 41 and 34 observed at the G409HUSZ and Ibit03014, respectively; and 39 and 43 genotypes of 6-, 7-, and 8-ploid out of the 48 and 54 observed ones at the Ibit11182 and the Ibit12692, respectively. However, the frequency distribution of these genotypes at each of the 4 loci did not fit the corresponding ones expected from a disomic or complete polysomic inheritance (Chi-square or multinomial goodness of fit p <0.001 or <0.0001), although the frequencies of many of the genotype classes fitted well to those expected from a polysomic inheritance. At the G409HUSZ, the observed frequencies of six (a to f arrow marked in **Figure 5A**) out of 19 genotypes were significantly underrepresented or overrepresented as compared to the corresponding expected ones under a hexasomic inheritance (Z-test p: 0.015 to <0.001). The genotypes expected from a complete preferential pairing of the homoeolog-types under bivalent configuration at each of the 4 loci were among those expected from a polysomic inheritance in the first category. The total frequencies of these full dual-sourced genotypes with proportions from both preferential and random pairing of the parental homoeolog-types, one, three, three, and nineteen genotypes at the G409HUSZ, the Ibit03014, the Ibit11182, and the Ibit12692, respectively (**Supplementary Tables S3**-**S6**), were used to estimate the frequency of disomic (or preferential pairing) inheritance (F_Di_) at the loci (excluding the partial dual-sourced genotypes). We derived the following formula: F_Di_ = (F_Ods_ - F_Epl_)/(1- F_Epl_) for estimation of the disomic inheritance frequency (detailed in **Supplementary Table S7**), where F_Ods_ is the observed total frequency of the full dual-sourced genotypes (their total counts or total numbers of all expected genotypes), and F_Epl_ is the expected total frequency of these genotypes from a polysomic inheritance. The estimated frequencies of disomic inheritance at the Ibit03014, the Ibit11182, the G409HUSZ, and the Ibit12692 were 0%, 2.72%, 14.52%, and 36.92%, respectively. At the Ibit03014, we also observed a rare spontaneous mutation event that nullified the signal of either one of the probes in a progeny to lead to an ‘X’ homoeolog-type in an AABBCX genotype (not charted).

The second category included one (at the G409HUSZ and the Ibit11182) or two (at the Ibit03014) hexaploid genotypes (‘I’ marked) carrying IDR ‘A’ or ‘C’ pairs, and one (at all but the Ibit12692) and three (at the Ibit12692) apparent aneuploid/dysploid genotypes (‘I/U’ marked) involving gametic genotypes having either IDR homoeolog-type pairs or partly unreduced homoeolog-types, just as those observed among F1 genotypes, but in lower frequencies. For example, the AAAAAC F2-genotype from the selfing of the parental AABBCC at the G409HUSZ should have come from the gametic AAC and AAA genotypes, the AAA of which could have arisen only from two A’s brought in the gamete from sister chromatids by double-reduction along with another ‘A’ on a homologous chromosome. The ABCCCCC F2-genotype (I/U) at the G409HUSZ could have come from a partly unreduced gametic genotype ABCC (‘CC’ by translocation or unbalanced segregation) and the CCC genotype carrying an IDR ‘C’ pair from the parental AABBCC genotype. The third category included apparent aneuploid and dysploid genotypes of locus-dependent classes and frequencies, as well as those observed among F1 genotypes. At the G409HUSZ, there were seven 5-ploid, twelve 7-ploid, and one 8-ploid, three (AAABBCC, AABBBCC, and AABCC) of which had frequencies comparable to some of the hexaploid genotypes in the first category. The high-frequency co-occurrence of the 5- and 7-ploid types with paired loss or gain by one ‘ploid’ from the hexaploid, especially the two (7 AABCC and 9 AABBBCC with a difference of one B pair) with statistically equal proportions, indicated that they were probably either dysploids or aneuploids of coupled chromosome-number changes in gametes, rather than mutant genotypes carrying spontaneous deletions and cis-duplications or due to unequal crossover of much lower frequencies. Similarly, at Ibit03014, there were 15 aneuploid/dysploid types, including eight 5-ploid, four 7-ploid, one 8-ploid, and two 9-ploid in comparatively low frequencies. At the Ibit11182 and the Ibit12692, the parental genotypes (ABBBBC and AAABBBC) themselves were 7-ploid and generated unexpectedly five 5-ploid and two 9-ploid, and four each of 5- and 9-ploid, respectively. The extra homeolog-type ‘ploid’ in the two parental genotypes was probably *trans* on a separate chromosome rather than a *cis*-duplication, as the latter would have led to only 6-ploid and 7-ploid genotypes of statistically equal frequencies, rather than the observed 6-ploid, 7-ploid, and 8-ploid genotypes in statistically equal proportions (14:13:12 and 16:14:13 types at the Ibit11182 and the Ibit12692, respectively) in the first category.

### 3.6 Effects of breeding selections on homoeolog-type genotypes

**Figure 6:**
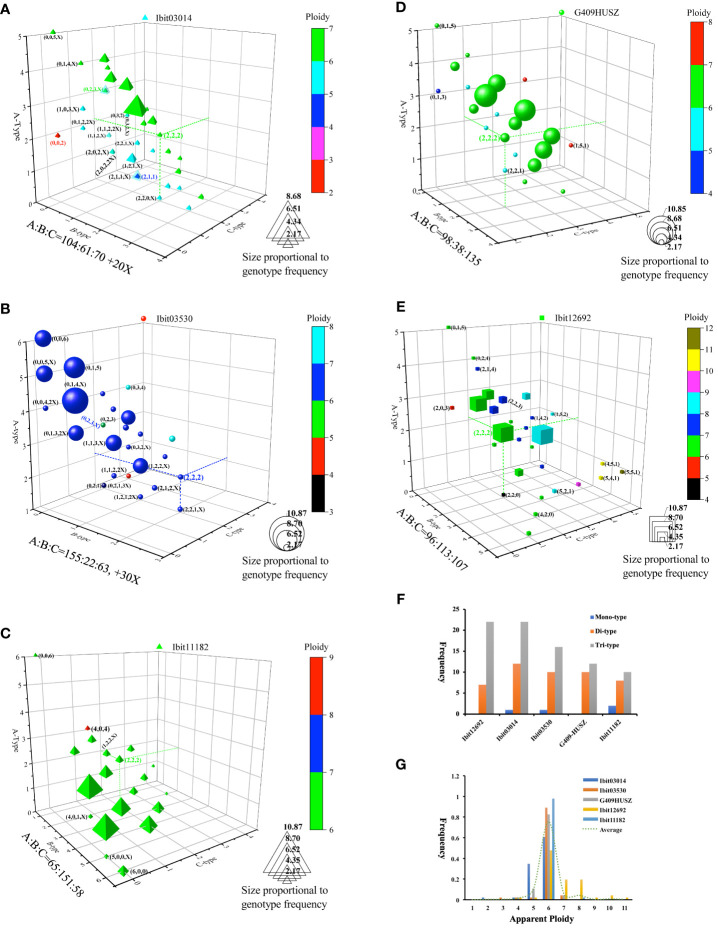
Effects of breeding selections on homoeolog-type genotypes in sweet potatoes. **(A–E)** 3D-graph summary of homoeolog-type genotypes at five selected loci in 46 sweetpotato lines from various cultivation regions. The observed 46 homoeolog-type genotypes at each of the loci were plotted on a 3D graph using the numbers of the A, B and C homoeolog-types in the so-called genotypes as coordinates. The color and size of the locus symbol for a genotype mark its apparent ploidy (total numbers of homoeolog-types) and frequency, respectively. The three dashed lines on each graph highlight coordinates of the evenly differentiated “AABBCC” genotype. The coordinates of some notable genotypes were listed in nearby parentheses (in B, C, and A order). Additions of X, 2X, or 3X at the end of the coordinates for some genotypes indicate one, two, or three additional homoeologs that were null-signaled to the A, B, and C homoeolog-type probes but detected by the corresponding control probes (i.e., control signal =A+B+C+X or 2X or 3X). **(F)** Comparisons of the frequencies of three genotype classes at each of the 5 loci. The mono-, di-, and tri-type genotypes refer to genotypes containing one (e.g., AAAAAA), two (e.g., AAABBB), or three (e.g., AABBCC) types of homoeolog-types, respectively. **(G)** Frequencies of the apparent ploidy of homoeolog-type genotypes at the 5 loci. The dashed trend line is the one connecting the averaged frequencies at each apparent ploidy (1 to 11).

We also sought to identify the effects of breeding selections on homoeolog-type genotypes by genotyping the 5 loci in 46 sweetpotato cultivars and lines from various cultivation regions (**Supplementary Data S11**). For trend visualization, we summarized the 46 genotypes at each of the loci on a 3D-plot, using numbers of the A, B, and C homoeolog-type in a genotype as the coordinates and the label size and color for frequencies and apparent ploidy of the genotype (**Figures 6A–E**). We also summarized the frequencies of genotypes with mono-, di-, and tri-type homoeolog-types and those of each observed apparent ploidy at the 5 loci (**Figures 6F, G**). These genotypes revealed locus-dependent selection biases. First, breeding selections favored genotypes with more of one homoeolog-type and reduction of tri-type (combinations of A, B, and C) to di (combinations of either two of the three homoeolog-types) or even monotype homoeolog-type composition at four of the loci. At the Ibit03014, about 41.3% of the genotypes had 3 to 5 A’s, including one count of an apparent ‘AA’ diploid type. At the Ibit03530, about 71.7% or 45.7% of the genotypes had 3 to 6 or 4 to 6 A’s, respectively, including three counts of the homogenized AAAAAA genotype. The genotypes at the Ibit11182 included those with 3 to 6 or 4 to 6 B’s at about 80.4% or 50.0%, respectively, two counts of the BBBBBB and one count of the AAAAAA genotype. At the G409HUSZ, about 58.7% of the genotypes had 3 to 5 C’s. In addition, the proportions of tri-type genotypes were triple and double those of the di-types at the Ibit12692 and the Ibit03014, respectively, but statistically equal to those of the di-types at the other 3 loci (**Figure 6F**). Consequently, such selection biases led to a predominant frequency for one of the three homoeolog-types among all the genotypes at each of four out of the 5 loci, i.e., in B, A, and C order at the Ibit03014 and the Ibit03530, in A, B, C order at the Ibit11182, in A, C, B order at the G409HUSZ, but statistically equal A, B, and C at the Ibit12692. Secondly, breeding selections favored one-directional change of ploidy in apparent aneuploid and dysploid genotypes at different loci. **Figure 6G** showed a bidirectional change of apparent ploidy among all the genotypes and skewedness toward one-directional change at different loci. The trend line from averaged frequencies at each ploidy fitted an almost perfect normal distribution with the mean at the hexaploid. The genotypes at the Ibit03014, the Ibit03503, and the G409HUSZ displayed distributions of 2-, 4-, 5-, 6-, and 7-ploid, 3-, 4-, 5-, 6 and 7-ploid, and 4-, 5-, 6-, and 7-ploid, respectively, with more aneuploids and dysploids of reduced ploidy. But genotypes at the Ibit12692 included 4-, 5-, 6-, 7-, 8-, 9-, 10-, and 11-ploid, having more aneuploids and dysploids of increased ploidy. At the Ibit11182, all genotypes except one at the 8-ploid remained at the hexaploid.

## 4 Discussion

To improve our understanding of the hexaploid sweetpotato, it is critical to clearly define its polyploid type and inheritance. Under the currently predominant “mode of origin” definition (Ramsey and Schemske, 2002; Mason and Wendel, 2020), the focal point of the debate on an allohexaploid or autohexaploid sweetpotato became whether it arose as an interspecies hybrid of *I. trifida* and its closely related species or an intraspecies varietal hybrid of *I. trifida*. This long-lasting debate stemmed partly from the confusing overlap of many cytogenetic and genetic characteristics of allopolyploids and autopolyploids. Pure allopolyploid and autopolyploid represent two ends of the polyploidy continuum. Varying progresses of postpolyploid diploidization and diversification resulted in many intermediates of both types having broadly overlapping cytogenetic and genetic characteristics (Ramsey and Schemske, 2002; Spoelhof et al., 2017; Mandakova and Lysak, 2018; Pele et al., 2018; Mason and Wendel, 2020; Mayrose and Lysak, 2021). Both an allopolyploid and an established autopolyploid could exhibit a polysomic inheritance with a low-level autosyndesis under a predominant bivalent configuration (Ramsey and Schemske, 2002; Mason and Wendel, 2020). Thus, these cytogenetic and genetic characteristics, if observed in the hexaploid sweetpotato, cannot be used to conclude either an auto- or allohexaploid sweetpotato, as they do not specifically correspond to either of them when the mode of the sweetpotato origin is uncertain. However, since we previously proposed a segmental allohexaploid sweetpotato based on a species hybridity (i.e., distinguishable homoeolog differentiation and a species hybridization phylogeny), we could test a null hypothesis that a segmental hexaploid sweetpotato should display a mixed inheritance as other known segmental allopolyploids (Shinohara et al., 2010; Jeridi et al., 2012; Leal-Bertioli et al., 2018; Mason and Wendel, 2020).

### 4.1 Genotyping three homoeolog-types by dqPCR

A mixed inheritance in allohexaploids involves segregations of both tri-type homoeologs from different subgenomes and bi-type homologs of the same subgenome under allosyndetic and autosyndetic bivalent formation, respectively. Segregations of tri-type homoeologs are of primary importance to mixed inheritance due to the implications of homoeolog exchanges (HEs) and associated high-frequency meiotic abnormalities. We thus sought to analyze segregations of three homoeolog-types that could mark all three corresponding homoeolog pairs in the hexaploid sweetpotato. To genotype such homoeolog-types at selected single-copy loci in sweetpotato, we initially attempted the genotyping-by-sequencing (GBS) approach but failed to get a consistent or statistically valid fitting of the homoeolog-type ratios. We later realized that there are two fatal problems in GBS applications in sweetpotato in addition to those of “overdispersion, allelic bias, and outlying observations” (Gerard et al., 2018). The first is the distortion of the marker-target proportions in genomic amplicon templates and sequencing libraries by exponential PCR amplifications. It is almost improbable to achieve co-linear amplifications of three genomic targets with very large proportional disparities, such as 1:5:0, 1:4:1, and 1:6:0, in a single PCR during template preparation and later library amplification. The second is its ineffectiveness to resolve genotypes having multiplexed target types with equivalent target ratios (e.g., 1:1:1 vs. 2:2:2, and 2:1:1 vs. 4:2:2, etc.), particularly those of aneuploids, dysploids, and euploids. For example, even without distortions of target proportions, a target-reads ratio of 1:1:1 should not be simply fitted to a 2:2:2 target genotype, as an eu-hexaploid genotype could not be simply assumed. We thus developed the LNA-FRET dqPCR genotyping method to overcome these genotyping problems in sweet potatoes.

Although the dqPCR method is low-throughput and it is difficult to develop multiple multiplexable probes at a given locus, its absolute quantification of target concentrations by digital counting of nano-partitions signaling the presence of probe-targets is independent of the PCR amplification kinetics, eliminating target-ratio distortion. It also allows to differentiate equivalent probe-target ratios for genotypes of different apparent ploidy, to properly call aneuploid and dysploid genotypes, and to detect null-signaled homoeolog-types. The dqPCR genotyping is of high specificity conferred by the loci-specific PCR primers and the target-specific LNA-FRET probes, and the results are highly reproducible and consistent. Additionally, multiplex-probe dqPCR genotyping of multiple loci using the same DNA sample eliminated quantitation errors from variations in DNA concentrations and contents of plastid and mitochondrial DNA.

### 4.2 The F2 population for segregation analyses

It has been extremely difficult to construct appropriate mapping populations of sufficient sizes from desired crosses in sweetpotato due to self-incompatibility and cross-incompatibility among various lines. The reported F2 population is currently the only one of its kind in sweet potatoes. The other partly comparable mapping population was the X18-S1 of very small sizes (142, 248 progenies) from the selfing of the Xushu-18 cultivar (Shirasawa et al., 2017; Yamamoto et al., 2020), which was a backcross progeny with incomplete pedigree information (Sheng et al., 1981). All other reported populations in sweetpotato were F1 hybrids (Yan et al., 2022). We evaluated the size adequacy of the genotyped F2 population for proper genotype representations by statistically equal ratios of the three homoeolog-types at a locus in the F2 population and in the parental R5 genotype, as random segregations and/or independent assortments of the three homoeolog-types from a parental genotype during the self-crosses should distribute the three homoeolog types by their original frequencies into progeny genotypes of sufficient numbers regardless of inheritance types. We initially genotyped 400 progenies but observed a statistically significant difference between the ratio of the homoeolog-types totaled from the 400 genotypes at a locus and that in corresponding pararenal genotypes. With additional genotyping of 149 progenies, the two ratios at each of the 4 loci became statistically equal. This F2 population size of 549 for statistically valid genotype representation is also in agreement with an earlier estimation of an adequate and practical F2 population size of 400 to 500 in sweetpotato (Jones, 1967).

### 4.3 A complex mixed-inheritance and biased breeding selections

The homoeolog-type genotypes of selected F1 progenies (**Figure 4**) at the 5 loci and segregations of the three homoeolog-types at 4 loci in the F2 population (**Figure 5**) exhibited two consistent inheritance patterns: 1. a mixed inheritance of disomic, polysomic, and intermediate types with locus-dependent frequencies of disomic inheritance, 2. high-frequency HEs, double-reduction recombination, and the generation of apparent aneuploidy or dysploidy with locus-dependent and possibly mating-type-dependent frequencies. In addition, the homoeolog-type genotypes at the 5 loci in a collection of extant sweetpotato cultivars revealed selection biases favoring homoeolog-type replacement and homogenization towards di- or mono-type genotypes at different loci. These inheritance patterns indicated that frequent HEs under biased selections could have resulted in partly or completely homogenized segments among otherwise homoeologous chromosomes in extant sweetpotato lines, which is consistent with the recently proposed mechanism of an allopolyploid evolving to a segmental one (Mason and Wendel, 2020).

The estimated frequencies of the disomic inheritance at the Ibit12692, the G409HUSZ, the Ibit11182, and the Ibit03014 in the F2 population, i.e., 36.92%, 14.52%, 2.72%, and 0%, respectively, should be underestimated as they did not include partial dual-sourced F2 genotypes having one gametic genotype derived from preferential pairing of homologous homoeolog-types. At the Ibit03014, although there was no contribution of disomic inheritance, the frequency distribution of the observed genotypes did not fit that of what was expected from a hexasomic inheritance (p-value:<0.0001) either, indicating an intermediate inheritance at the locus. Please note that the multinomial goodness of fit had to be used for fitting genotype frequencies at three of the 4 loci in the F2 population because there were fewer than five counts for multiple genotypes (2, 9, and 16 at the Ibit03014, the Ibit11182, and the Ibit12692, respectively). A wrong use of the chi-square test for fitting the genotype frequencies at the Ibit03014 would have yielded a misleading p-value of 0.232, leading to a false conclusion of fitting to a hexasomic inheritance, which could have happened if the two genotypes with very low frequencies (1 AAAACC and 3 BBBBBB) were not detected by an appropriate method or in a population of insufficient size. The low frequency of disomic inheritance (2.72%) at the Ibit11182 could have resulted from a vastly underrepresented dual-sourced 8-ploid genotype (AABBBBCC), as its observed count ratio to the other two dual-sourced ones (ABBBBCC and BBBBCC), 9:58:37, was way out of proportion to those expected under either disomic or polysomic inheritance, 1:2:1 and 50:39:3, respectively. This under-representation was probably due to the much-reduced viability of either parental aneuploid or dysploid gametic genotypes (i.e., 4- and 4-ploid or 3- and 5-ploid) or the sporophyte of the genotype. We indeed observed F2 progenies of low viability or growth deficiency in about 10% of the germinated ones, among which some died off after senescence of cotyledons, and some others displayed extremely stunned growth (~10 cm for 2 years) or severe disease susceptibility. Additionally, the frequencies of the full dual-sourced F1 genotypes among the Resisto-mothered ones at the Ibit12692, the G409HUSZ, the Ibit11182, and the Ibit03014, i.e., 12, 7, 1, and 3 out of 17, respectively (**Supplementary Table S2**), were consistent in a size order with the estimated frequencies of disomic inheritance at the corresponding loci in the F2 population. This indicates that the frequency of disomic inheritance in the F1 generation may be similarly dependent on locus, although it could not be accurately estimated due to the small population size. The observation of a mixed inheritance with locus-dependent frequencies of the disomic inheritance at the 4 loci indicates that the extent of a mixed inheritance (or proportions of disomic, polysomic, and intermediate inheritance types) may also vary by chromosomes in the sweetpotato genome, which must be further assessed by appropriate genome-wide segregation analyses.The frequencies complementary to those of disomic inheritance at the 4 loci in the F2 population were those (>63%) of polysomic and intermediate inheritance types, which implied a very high frequency of HEs under allosyndetic bivalent configuration. Additionally, detections of low-frequency genotypes indicative of double reduction and of either double reduction or unreduced gametes at three and 4 loci in the F1 and F2 populations, respectively, indicated a low frequency of HEs under meiotic multivalent configuration. These two patterns are consistent with the earlier cytological observations of a predominant bivalent and low-frequency multivalent configuration (Magoon et al., 1970). The double-reduction recombination could also have contributed to the observed mono-type genotypes of homogenized homoeolog-types at some loci in several F2 lines and cultivars, as it could slightly increase the homogeneity of a locus (Huang et al., 2019) by bringing two identical sister-chromatid segments into a single gamete. The observed frequencies of 1 to 3 out of 549 for the IDR genotypes at the 4 loci in the F2 population may well be an underestimation since gametes carrying IDR homoeolog-type pairs could fertilize with regular gametes to yield expected genotypes with undistinguishable IDR pairs. This assessment was corroborated by the relative high frequencies of the F1 IDR genotypes, particularly at Ibit11182. The detection of the IDR genotypes in this study provided evidence, for the first time, to reject a long-standing assumption of no double-reduction recombination in sweetpotato and the deduced allele segregation ratios under the assumption (Jones, 1967).

We also demonstrated a consistent existence of varying classes and frequencies of apparent aneuploid and dysploid genotypes unexpected from either disomic or polysomic inheritances at three and 4 loci in the F1 and F2 populations, respectively. These apparent aneuploid/dysploid (indistinguishable by genotypes) genotypes were probably derived from partially unreduced gametes due to translocations or unbalanced segregations rather than simple copy-number changes by deletions or *cis*-duplications on the original chromosomes. This conclusion is supported by their much higher occurrence frequencies and larger class numbers, especially at the G409HUSZ, than those expected from spontaneous mutations or unequal crossovers, and by the co-occurrence of genotypes with paired loss or gain of one or two homoeolog-types as compared with corresponding parental genotypes. The frequencies of apparent aneuploid/dysploid genotypes at the G409HUSZ and the Ibit03014 were 9.67% and 5.1%, respectively, which were either close to the 11.5% average or at the low end of those in neoallopolyploids (Ramsey and Schemske, 2002). The parental genotypes at the Ibit11182 and the Ibit12692 themselves were 7-ploid aneuploids/dysploids, and they were segregated into expected hexaploids and aneuploids/dysploids of expected 7- and 8-ploids and unexpected 5- and 9-ploids in large class numbers and relatively high frequencies (>95%). Additionally, the bi-directional deviation of apparent ploidy from hexaploidy among all genotypes at the 5 loci in the 46 cultivars and lines displayed a normal frequency distribution that matched those in a neopolyploid (Ramsey and Schemske, 2002). These neoallopolyploid-like features indicated that sweet potato is far from a stable or established hexaploid. All the above observed features demonstrated an evolving segmental allohexaploid sweetpotato featuring a mixed inheritance with high-frequency HEs and meiotic aberrations and impacted by biased breeding selections.

## Data availability statement

The original contributions presented in the study are included in the article/**Supplementary Material**, further inquiries can be directed to the corresponding author.

## Author contributions

MG: Conceptualization, Data curation, Formal analysis, Funding acquisition, Investigation, Methodology, Project administration, Resources, Supervision, Validation, Writing – original draft, Writing – review & editing. TH: Data curation, Formal analysis, Investigation, Validation, Writing – review & editing. GN: Formal analysis, Investigation, Resources, Validation, Writing – review & editing. JM: Data curation, Formal analysis, Investigation, Resources, Writing – review & editing. WD: Investigation, Project administration, Resources, Writing – review & editing.
