# Supplementary material for: A locus-dependent mixed inheritance in the segmental allohexaploid sweetpotato (Ipomoea batatas [L.] Lam)
Source: Front Plant Sci. 2024 May 28;15:1398081. doi: 10.3389/fpls.2024.1398081 (PMC11165125; doi:10.3389/fpls.2024.1398081)
Supplement: Supplementary file 1 [file DataSheet_1.pdf]

S1 Table. Summary of dqPCR primers and LNA-FRET probes for five selected "single-copy" loci in sweetpotato.

Loci	Primers/Probes		Sequence	Tm °C Match	Tm °C Mismatch	dqPCR Tm °C
Ibit03014	PCR Primers	Forward	AGGCTATTGATCTACTAGAAATGCTA	57.3		60
		Reverse	TTCTTTGTGCGTTCATCAAGAA	57.1		
	Probes	A	(5'6-FAM)TA+T+G+CTATACA+C+A+TATGG(3'IABkFQ)	66.1	49.6	
		B	(5'SUN)CTG+C+C+AA+T+A+GTTTC(3'IABkFQ)	67.9	42.8	
		C	(5'TEX615)TC+C+C+TG+T+TT+CT(3'IAbRQSp)	65.7	45.9	
		Control	(5'Cy5)CAT+G+A+ACCA+A+G+CAC(3'IAbRQSp)			
Ibit03530	PCR Primers	Forward	CTAGTGTCGCACCGAGAAC	58.0		60
		Reverse	GCTGTTCTTCATTCATAGGCA	56.3		
	Probes	A	(5'6-FAM)AGTT+A+G+TC+T+T+TGCCA(3'IABkFQ)	65.1	53.3	
		B	(5'SUN)TT+A+G+TC+T+C+TGCCA(3'IABkFQ)	66.5	50.7	
		C	(5'TEX615)AGTT+A+C+TCT+T+TGC+CA(3'IAbRQSp)	65.3	44.8	
		Control	(5'Cy5)TG+G+GAT+G+CTA+CA(3'IAbRQSp)			
Ibit-G409HUSZ	PCR Primers	Forward	TTAGTCCAATCACGAAGATCATCC	56.2		60
		Reverse	AATTGATGCAGTTATAATAACTCATTCT	58.4		
	Probes	AB	(5'6-FAM)TGAT+T+CA+CT+G+ATTAT+CTAA(3'IABkFQ)	66.0		
		B	(5'SUN)TT+C+ATA+C+GA+GTA+CA(3'IABkFQ)	65.2	31.8	
		C	(5'TEX615)AGT+G+AT+T+CT+CTAA+ACA(3'IAbRQSp)	65.0		
		Control	(5'Cy5)TG+C+TC+CT+G+CC(3'IAbRQSp)	67.4		
Ibit11182	PCR Primers	Forward	CCTTTTGGATATACTAGAAAGGATGTTC	58.0		60
		Reverse	CCTGAAGACGTATGTTGAAATCCATC	60.2		
	Probes	A	(5'6-FAM)ACAC+C+A+AA+G+AA+CT(3'IABkFQ)	65.2	53.9	
		B	(5'SUN)AGC+C+A+GC+A+ACC(3'IABkFQ)	65.8	51.5	
		C	(5'TEX615)TCAA+C+T+CTATA+A+CT+CT(3'IAbRQSp)	65		
		Control	(5'Cy5)TG+G+A+CTGG+A+GG(3'IAbRQSp)	68.6		
Ibit12692	PCR Primers	Forward	AGGTTGAATCAACACTGGCAA	58.3		64
		Reverse	AGAGGATTAACACCTGCCTGTC	59.8		
	Probes	A	(5'6-FAM)AG+A+C+GT+G+T+GATAC(3'IABkFQ)	65.2	50.1	
		B	(5'SUN)AGA+C+GT+G+C+GAT(3'IABkFQ)	65.4	50.8	
		C	(5'TEX615)AG+A+T+GTG+T+GA+TACT(3'IAbRQSp)	65.7	55.7	
		Control	(5'Cy5)AA+GAATA+CAA+CTT+G+A+GA(3'IAbRQSp)	68.1		

3'IABkFQ and 3'IAbRQSp: 3' Iowa Black® FQ and 3' Iowa Black® RQ quencher, respectively.
The 5'6-FAM, 5'SUN, 5'TEX615 and 5'Cy5: 5' labeled Fluorescein, Sun, Tex615, and Cy5 fluorescent dyes, respectively. "+": locked base after the sign. The Ibit03014: predicted PP2A regulatory subunit TAP46-like gene. The Ibit03530: peptidyl-prolyl cis-trans isomerase CYP23-like gene. The G409HUSZ: putative hydrolase C777.06c gene. Th Ibit11182: unknown gene. The Ibit12692: inner membrane protein PPF-1like gene.
