# Supplementary material for: A locus-dependent mixed inheritance in the segmental allohexaploid sweetpotato (Ipomoea batatas [L.] Lam)
Source: Front Plant Sci. 2024 May 28;15:1398081. doi: 10.3389/fpls.2024.1398081 (PMC11165125; doi:10.3389/fpls.2024.1398081)
Supplement: Supplementary file 2 [file DataSheet_2.pdf]

S2 Table. Summary of observed and expected Resisto-mothered F1 genotypes and their frequencies at the three loci.

AAAABB x AABBCC(♀) At Ibit03014				ABBBCC x AAABCC(♀) At G409HUSZ				AAABBBC x AABBBC(♀) At Ibit12692			
F1 Genotype	Observed (/17)	^{R}Expected Freq. (%)	^{D}Expected Freq.(%)	F1 Genotype	Observed (/17)	^{R}Expected Freq.(/400)	^{D}Expected Freq.(%)	F1 Genotype	Observed (/17)	^{R}Expected Freq.(/3500)	^{D}Expected Freq. (/12)
AAAAAB	0	2.5	0	AAABBB	0	10	0	**AAABBB[2]**	1	82	1
AAAAAC	0	2.5	0	AABBBB	0	3	0	**AAABBC[2]**	2	111	1
AAAABB[1]	0	10	0	ABBBBC[1]	0	6	0	AAAABC[1]	1	24	0
AAAACC	0	2.5	0	AABBBC[1]	1	42	0	AAAABB[1]	1	33	0
**AAABBC[2]**	3	20	100	**ABBBCC[2]**	1	42	25	AAAAAB[1]	0	3	0
AAABCC	4	12.5	0	BBBBCC	0	1	0	AAAAAC	1	1	0
AAAABC	0	12.5	0	AAAABB	0	3	0	**AABBBB[2]**	0	66	1
AAABBB	1	10	0	AAABBC[1]	2	42	0	**AABBBC[2]**	4	148	2
AABBBC[1]	3	12.5	0	**AABBCC[2]**	3	90	50	ABBBBB[1]	1	15	0
AABBCC[1]	3	7.5	0	AAAABC[1]	0	6	0	ABBBBC[1]	1	60	0
AABBBB	1	2.5	0	AAAACC	0	1	0	**AABBCC[2]**	0	66	1
ABBBCC	0	2.5	0	**AAABCC[2]**	3	42	25	AAABCC[1]	0	27	0
ABBBBC[1]	1	2.5	0	AABCCC[1]	3	42	0	AAAACC	0	3	0
ABBCCC	1	0	0	ABBCCC[1]	0	42	0	ABBBCC[1]	0	45	0
				BBBCCC[1]	0	6	0	BBBBBB	0	1	0
BBCCCC x AABBBB(♀) At Ibit11182				ABCCCC	1	10	0	BBBBBC	0	6	0
AABBBC	0	4	0	BBCCCC	0	3	0	BBBBCC	0	9	0
ABBBBC[1]	0	12	0	AACCCC	0	3	0	AAABBBB[1]	0	264	0
BBBBBC	1	4	0	AAACCC[1]	1	6	0	**AAABBBC[2]**	4	592	2
AABBCC[1]	3	12	0	AAAABCC	1	0	0	AAAABBC[1]	0	240	0
**ABBBCC**[2]	1	36	100	ABBCC	1	0	0	AAAABBB[1]	0	180	0
BBBBCC[1]	1	12	0					AAAAABB[1]	0	36	0
AABCCC	1	4	0					AAAAABC[1]	0	24	0
ABBCCC [1]	2	12	0					**AAABBCC[2]**	0	264	2
BBBCCC	0	4	0					AAAABCC[1]	0	60	0
AABBBB	2	0	0					AAAAACC	0	4	0
ABBBBB	1	0	0					AABBBBB	0	108	0
AABBBBBB	1	0	0					**AABBBBC[2]**	1	444	1
AABBBBC	1	0	0					**AABBBCC[2]**	0	328	1
ABBBBCC	2	0	0					ABBBBBB	0	12	0
BBBBC	1	0	0					ABBBBBC	0	96	0
								ABBBBCC[1]	0	132	0
								BBBBBBC	0	4	0
								BBBBBCC	0	12	0

^{R}: Under complete random pairing of all the homoeolog-types. ^{D}: Under preferential pairing of the homologous homoeolog-types (i.e., AA, or BB, or CC first). [1]: Involving one gametic genotype derived from preferential paring of the homologous homoeolog-types (i.e., AA, or BB, or CC). [2]: Involving both gametic genotypes derived from preferential paring of the homologous homoeolog-types (i.e., AA, or BB, or CC) or from random pairing of all the 6 or 7 homoeolog-types.
