# Supplementary material for: A locus-dependent mixed inheritance in the segmental allohexaploid sweetpotato (Ipomoea batatas [L.] Lam)
Source: Front Plant Sci. 2024 May 28;15:1398081. doi: 10.3389/fpls.2024.1398081 (PMC11165125; doi:10.3389/fpls.2024.1398081)
Supplement: Supplementary file 3 [file DataSheet_3.pdf]

S3 Table. Segregation of the homoeolog-types at the G409HUSZ in the F2 population from self-crossing of the "AABBCC" parental genotype.

Genotypes	Observed Counts	Under Disomic Inheritance	Under Random Paring of the Homoeolog-Types in Bivalent Configuration				
		Expected Counts (Freq.)	Expected Counts (Freq.)	Chi-Square Goodness of Fit Test (Z-value based)			
				[a]z-value	[b]p-value	[c]Dir.	χ^2 test
AAAABB	10	0	8 (1/64)	0.83	0.409	–	
AAAABC	17	0	15 (1/32)	0.39	0.697	–	
AAAACC	6	0	8 (1/64)	-0.62	0.532	–	
AAABBB	6	0	15 (1/32)	-2.45	0.014	↓	
AAABBC[1]	60	0	46 (3/32)	2.09	0.036	↑	
AAABCC[1]	41	0	46 (3/32)	-0.84	0.402	–	
AAACCC	9	0	15 (1/32)	-1.68	0.094	–	
AABBBB	4	0	8 (1/64)	-1.35	0.177	–	χ^2 = 90.88
AABBBC[1]	39	0	46 (3/32)	-1.15	0.252	–	p-Value:
**AABBCC[2]**	138	495 (1.0)	77 (5/32)	7.5	<0.001	↑	<0.001
AABCCC[1]	43	0	46 (3/32)	-0.53	0.597	–	(DF=18)
AACCCC	1	0	8 (1/64)	-2.44	0.015	↓	
ABBBBC	5	0	15 (1/32)	-2.71	0.007	↓	
ABBBCC[1]	36	0	46 (3/32)	-1.61	0.108	–	
ABBCCC[1]	51	0	46 (3/32)	0.7	0.481	–	
ABCCCC	6	0	15 (1/32)	-2.45	0.014	↓	
BBBBCC	6	0	8 (1/64)	-0.62	0.532	–	
BBBCCC	8	0	15 (1/32)	-1.93	0.053	–	
BBCCCC	9	0	8 (1/64)	0.46	0.643	–	
**Unexpected**							
AAAAAC	1	0	0	Carrying an IDR pair of 'A' from an 'AAA' gametic genotype			
ABCCCCC	1	0	0	Carrying an IDR pair of 'C' from 'CCC' or 'A(or B)CCC' gametic genotype, and/or a pair of 'C' from partially unreduced gametes of several possible tetraploid or triploid genotypes.			
AAAABBB	2	0	0				
AAAABBC	1	0	0				
AAAABCC	1	0	0				
AAABB	2	0	0				
AAABBBC	2	0	0				
AAABBCC	8	0	0				
AAABBCCC	1	0	0				
AAABCCC	1	0	0				
AABBB	2	0	0				
AABBBBC	1	0	0	Aneuploidy/Dysploidy			
AABBBCC	9	0	0				
AABBC	3	0	0				
AABBCCC	3	0	0				
AABCC	7	0	0				
AABCCCC	1	0	0				
ABBBC	2	0	0				
ABBBCCC	2	0	0				
ABBCC	1	0	0				
ABBCCCC	1	0	0				
BBCCC	2	0	0				

[a]: Z-test for comparing each observed genotype frequency with its corresponding expected frequency.

[b]: P-value based on the Z-test.

[c]: Indicates whether an observed proportion is over or under represented compared to its corresponding expected proportion.

[1]: Involving one gametic genotype that may be derived from preferential pairing.

[2]: Involving one gametic genotype that were derived from preferential pairing.

IDR: Identical-by-double-reduction.
