# Supplementary material for: A locus-dependent mixed inheritance in the segmental allohexaploid sweetpotato (Ipomoea batatas [L.] Lam)
Source: Front Plant Sci. 2024 May 28;15:1398081. doi: 10.3389/fpls.2024.1398081 (PMC11165125; doi:10.3389/fpls.2024.1398081)
Supplement: Supplementary file 4 [file DataSheet_4.pdf]

**S4 Table. Segregation of the homoeolog-types at the Ibit03014 in the F2 population from self-crossing of the "AABBBC" parental genotype.**

| Genotypes | Observed Counts | [D]Expected Counts (Freq.) | [R]Expected Counts (Freq.) | Note: |
|---|---|---|---|---|
| AAAABB | 13 | 0 | 12(9/400) | Multinomial goodness of fit (Monte Carlo method) to the [R]Expected: p-value: <0.0001 at 5% significance level, and with 10000 simulations. |
| AAAABC | 13 | 0 | 8(6/400) | |
| AAAACC | 1 | 0 | 1(1/400) | |
| AAABBB[1] | 46 | 0 | 47(36/400) | |
| AAABBC[1] | 64 | 0 | 62(48/400) | |
| AAABCC[1] | 20 | 0 | 16(12/400) | |
| **AABBBB[2]** | 70 | 130(0.25) | 54(42/400) | |
| **AABBBC[2]** | 106 | 259(0.50) | 119(92/400) | |
| **AABBCC[2]** | 43 | 130(0.25) | 54(42/400) | |
| ABBBBB[1] | 19 | 0 | 16(12/400) | |
| ABBBBC[1] | 56 | 0 | 62(48/400) | |
| ABBBCC[1] | 48 | 0 | 47(36/400) | |
| BBBBBB | 3 | 0 | 1(1/400) | |
| BBBBBC | 6 | 0 | 8(6/400) | |
| BBBBCC | 11 | 0 | 12(9/400) | |
| AABBCX | 1 | 0 | 0 | Carrying a null-signaled homoeolog-type (X) due to a spontaneous mutation(s) in either of the probe-target regions |
| **Unexpected** | | | | |
| AAAAAB | 1 | 0 | 0 | Carrying an identical-by-double-reduction pair of 'A' from an 'AAA' gametic genotype. |
| ABBCCC | 1 | 0 | 0 | Carrying an identical-by-double-reduction pair of 'C' from an 'CCC' gametic genotype. |
| AAAAABC | 1 | 0 | 0 | Carrying an identical-by-double-reduction pair of 'A' from a 'AAA' or 'AAAB(or C)' gametic genotype, and/or a pair of 'A' from partially unreduced gametes of several possible tetraploid or triploid-type genotypes. |
| AAABBBB | 1 | 0 | 0 | Aneuploidy/Dysploidy |
| AAABBBBBB | 1 | 0 | 0 | |
| AAABBCC | 1 | 0 | 0 | |
| AAABC | 3 | 0 | 0 | |
| AABBB | 1 | 0 | 0 | |
| AABBBBB | 1 | 0 | 0 | |
| AABBBBCC | 1 | 0 | 0 | |
| AABBBCC | 2 | 0 | 0 | |
| AABBBCCCC | 1 | 0 | 0 | |
| AABBC | 2 | 0 | 0 | |
| ABBBB | 2 | 0 | 0 | |
| ABBBC | 5 | 0 | 0 | |
| ABBCC | 2 | 0 | 0 | |
| BBBBC | 2 | 0 | 0 | |
| BBBCC | 1 | 0 | 0 | |

[D]: Preferential Pairing (AA, BB first) under a bivalent configuration

[R]: Random Paring of the Homoeolog-Types under a bivalent configuration

[1]: Involving one gametic genotype that may be derived from preferential pairing.

[2]: Involving one gametic genotype that were derived from preferential pairing.
