# Supplementary material for: A locus-dependent mixed inheritance in the segmental allohexaploid sweetpotato (Ipomoea batatas [L.] Lam)
Source: Front Plant Sci. 2024 May 28;15:1398081. doi: 10.3389/fpls.2024.1398081 (PMC11165125; doi:10.3389/fpls.2024.1398081)
Supplement: Supplementary file 5 [file DataSheet_5.pdf]

S5 Table. Segregation of the homoeolog-types at the Ibit11182 in the F2 population from self-crossing of the "ABBBBCC" genotype.

Genotypes	Observed Counts	[D]Expected Counts (Freq.)	[R]Expected Counts (Freq.)	Notes
AABBBB	8	0	1 (36/30625)	Multinomial goodness of fit test (Monte Carlo method) to the [R]Expected Counts (Freq.)<br><br>p-value: <0.0001<br>at 5% Significance level, and with 10000 simulations.
AABBBC	22	0	2(96/30625)	
AABBCC	12	0	1(76/30625)	
ABBBBB	6	0	1(48/30625)	
ABBBBC[1]	27	0	4(208/30625)	
ABBBCC[1]	48	0	4(248/30625)	
AABCCC	1	0	0(16/30625)	
ABBCCC[1]	28	0	2(88/30625)	
AACCCC	0	0	0(1/30625)	
ABCCCC	3	0	0(8/30625)	
BBBBBB	3	0	0(16/30625)	
BBBBBC[1]	16	0	2(96/30625)	
**BBBBCC[2]**	37	135(0.25)	3(176/30625)	
BBBCCC[1]	14	0	2(96/30625)	
BBCCCC	10	0	0(16/30625)	
AABBBBB	6	0	3(192/30625)	
AABBBBC[1]	19	0	15(832/30625)	
AABBBCC[1]	18	0	17(992/30625)	
ABBBBBB	3	0	3(176/30625)	
ABBBBBC[1]	20	0	21(1216/30625)	
**ABBBBCC[2]**	58	270(0.5)	39(2216/30625)	
AABBCCC[1]	10	0	6(352/30625)	
ABBBCCC[1]	28	0	21(1216/30625)	
AABCCCC	0	0	1(32/30625)	
ABBCCCC	7	0	3(176/30625)	
BBBBBBB	0	0	1(32/30625)	
BBBBBBC[1]	6	0	6(352/30625)	
BBBBBCC[1]	26	0	17(992/30625)	
BBBBCCC[1]	26	0	15(832/30625)	
BBBCCCC	7	0	3(192/30625)	
AABBBBBB	0	0	5(256/30625)	
AABBBBBC[1]	6	0	27(1536/30625)	
**AABBBBCC[2]**	9	135(0.25)	50(2816/30625)	
ABBBBBBB	1	0	2(128/30625)	
ABBBBBBC[1]	6	0	25(1408/30625)	
ABBBBBCC[1]	12	0	70(3968/30625)	
AABBBCCC[1]	4	0	27(36/30625)	
ABBBBCCC[1]	13	0	59(1536/30625)	
AABBCCCC	0	0	5(256/30625)	
ABBBCCCC	5	0	14(768/30625)	
BBBBBBBB	0	0	0(16/30625)	
BBBBBBBC	2	0	5(256/30625)	
BBBBBBCC	4	0	21(1216/30625)	
BBBBBCCC	7	0	27(1536/30625)	
BBBBCCCC	2	0	10(576/30625)	
**Unexpected**				
AAABBB	1	0	0	Carrying an identical-by-double-reduction pair of 'A' from an 'AAB' gametic genotype
AAABBBBBC	1	0	0	Carrying either an identical-by-double-reduction pair of 'A' or a pair of 'A' in a partially unreduced gamete of either tetra- or pentaploid type.
AABBBBBBC	1	0	0	Aneuploidy/Dysploidy
AABBBBBCC	1	0	0	
ABBCC	1	0	0	
ACCCC	1	0	0	
BBBBC	1	0	0	
BBBCC	1	0	0	
BBCCC	1	0	0	

[D]: Preferential Pairing (BB, CC first, and random pairing with the extra A) under a bivalent configuration
[R]: Random Paring of the Homoeolog-Types under a bivalent configuration
[1]: Involving one gametic genotype that may be derived from preferential pairing.
[2]: Involving one gametic genotype that were derived from preferential pairing.
