# Supplementary material for: A locus-dependent mixed inheritance in the segmental allohexaploid sweetpotato (Ipomoea batatas [L.] Lam)
Source: Front Plant Sci. 2024 May 28;15:1398081. doi: 10.3389/fpls.2024.1398081 (PMC11165125; doi:10.3389/fpls.2024.1398081)
Supplement: Supplementary file 6 [file DataSheet_6.pdf]

S6 Table. Segregation of the homoeolog types at the Ibit12692 in the F2 population from self-crossing of the "AAABBBC" parental genotype.

Genotypes	Observed Counts	[D]Expected Counts (Freq.)	[R]Expected Counts (Freq.)	Notes
AAAAAA	0	0	0 (1/30625)	Multinomial goodness of fit test (Monte Carlo method)
AAAAAB[1]	4	0	0(18/30625)	p-value: <0.0001
**AAAABB[2]**	9	15 (0.0278)	2(99/30625)	At 5% Significance level, and with 10000 simulations:
AAAAAC	1	0	0(6/30625)	
AAAABC[1]	7	0	1(72/30625)	
**AAABBC[2]**	36	30(0.0556)	4(222/30625)	
**AAABBB[2]**	37	30(0.0556)	3(164/30625)	
**AABBBC[2]**	47	30(0.0556)	4(222/30625)	
**AABBBB[2]**	25	15 (0.0278)	2(99/30625)	
ABBBBC[1]	17	0	1(72/30625)	
ABBBBB[1]	4	0	0(18/30625)	
AAAACC	2	0	0(9/30625)	
AAABCC[1]	6	0	1(54/30625)	
**AABBCC[2]**	24	15 (0.0278)	2(99/30625)	
ABBBCC[1]	12	0	1(54/30625)	
BBBBCC	4	0	0(9/30625)	
BBBBBC	3	0	0(6/30625)	
BBBBBB	0	0	0(1/30625)	
AAAAAAB	0	0	0(24/30625)	
AAAAAAC	0	0	0(8/30625)	
AAAAABB[1]	3	0	5(288/30625)	
AAAAABC[1]	2	0	4(216/30625)	
**AAAABBB[2]**	19	30(0.0556)	16(888/30625)	
**AAAABBC[2]**	21	30(0.0556)	21(1224/30625)	
**AAABBBC[2]**	54	89(0.1667)	37(2104/30625)	
**AAABBBB[2]**	22	30(0.0556)	16(888/30625)	
**AABBBBC[2]**	32	30(0.0556)	21(1224/30625)	
AABBBBB	3	0	5(288/30625)	
ABBBBBC	8	0	4(216/30625)	
AAAAACC	0	0	0(24/30625)	
AAAABCC[1]	6	0	5(288/30625)	
**AAABBCC[2]**	22	30(0.0556)	16(888/30625)	
**AABBBCC[2]**	18	30(0.0556)	16(888/30625)	
ABBBBCC[1]	6	0	5(288/30625)	
BBBBBCC	0	0	0(24/30625)	
ABBBBBB	1	0	0(24/30625)	
BBBBBBC	0	0	0(8/30625)	
AAAAAABB	2	0	3(144/30625)	
AAAAAABC	0	0	2(96/30625)	
AAAAABBB	3	0	15(864/30625)	
AAAAABBC	0	0	20 (1152/30625)	
**AAAABBBB[2]**	6	15 (0.0278)	28(1584/30625)	
**AAAABBBC[2]**	11	30(0.0556)	62(3552/30625)	
**AAABBBBC[2]**	15	30(0.0556)	62(3552/30625)	
AAAAAACC	0	0	0(16/30625)	
AAAAABCC	2	0	5(288/30625)	
**AAAABBCC[2]**	8	15 (0.0278)	28(1584/30625)	
**AAABBBCC[2]**	14	30(0.0556)	46(2624/30625)	
AAABBBBB[1]	1	0	15(864/30625)	
AABBBBBC[1]	1	0	20(1152/30625)	
**AABBBBCC[2]**	12	15 (0.0278)	28(1584/30625)	
AABBBBBB	4	0	3(144/30625)	
ABBBBBBC[1]	0	0	2 (96/30625)	
ABBBBBCC[1]	1	0	5(288/30625)	
BBBBBBCC	0	0	0(16/30625)	
**Unexpected**				
AABBCCCC	1	0	0	Carrying two pairs of "C" from either IDR, or partially unreduced gamete
AAABBBCCC	1	0	0	Carrying one pairs of "C" from either IDR, or partially unreduced gamete
AAAABBCCC	1	0	0	
AAAAABBBC	1	0	0	
AAAABBBCC	2	0	0	
AAABBBBBC	1	0	0	Aneuploidy/Dysploidy
AAABBBBCC	1	0	0	
AAABB	1	0	0	
AABBB	3	0	0	
AABBC	1	0	0	
BBBCC	1	0	0	

[R]: Under a random paring of the homoeolog-types in bivalent configuration.
[D]: Under autosyndesis (AA, BB pairing) and allosyndesis (AB or AC) in bivalent configuration and random distribution of the unpaired one.
[1]: Involving one gametic genotype that may be derived from preferential pairing.
[2]: Involving one gametic genotype that were derived from preferential pairing.
IDR: Identical-by Double-Reduction.
