# Supplementary material for: A locus-dependent mixed inheritance in the segmental allohexaploid sweetpotato (Ipomoea batatas [L.] Lam)
Source: Front Plant Sci. 2024 May 28;15:1398081. doi: 10.3389/fpls.2024.1398081 (PMC11165125; doi:10.3389/fpls.2024.1398081)
Supplement: Supplementary file 7 [file DataSheet_7.pdf]

S7 Table. Estimation of the frequency for disomic inheritance (F_{Di}) based on full dual-sourced homoeolog-type genotypes at the four loci in the F2 population.

	At the G409HUSZ	At the Ibit03014	At the Ibit11182	At the Ibit12692
N	495	519	540	535
Tds	138	219	104	410
F_{Ods}	0.2788	0.4220	0.1926	0.7664
F_{Epl}	0.1563	0.4400	0.1700	0.6296
F_{Di}	0.1452	0	0.0272	0.3692
$F_{Di} = (F_{Ods} - F_{Epl}) / (1 - F_{Epl})$				

N: Total counts of expected genotypes, excluding those of unexpected abnormal ones,
Tds: Total observed full dual-sourced genotypes
F_{Ods} = Frequency of the total observed dual-sourced genotypes (Tds/N)
F_{Epl}: Frequency of the total dual-sourced genotypes expected from a complete random pairing of the homoeolog-types (Allosyndetic bivalent paring).

Derivation of the formula:
F_{Di} x N (disomic proportion) + N(1-F_{Di})F_{Epl} (polysomic proportion) = Total observed dual-sourced genotypes
Thus, $F_{Di} + F_{Epl} - F_{Di} \times F_{Epl}$ =Total Observed Dual-sournced Genotypes/N
$F_{Di} + F_{Epl} - F_{Di} \times F_{Epl} = F_{Ods}$
$F_{Di} (1-F_{Epl}) = F_{Ods} - F_{Epl}$
So, $F_{Di} = (F_{Ods} - F_{Epl}) / (1-F_{Epl})$
